# Tranexamic Acid as a Successful Therapy in Turner Syndrome With Recurrent Overt Gastrointestinal Bleeding due to Small Intestinal Venous Anomalies

**DOI:** 10.14309/crj.0000000000000961

**Published:** 2023-01-20

**Authors:** Pongtawat Lertwilaiwittaya, Frederick H. Weber

**Affiliations:** 1 Tinsley Harrison Internal Medicine Residency Program, Department of Medicine, University of Alabama at Birmingham, Birmingham, AL; 2 Department of Genetics, University of Alabama at Birmingham, Birmingham, AL; 3 Department of Medicine, Faculty of Medicine Siriraj Hospital Mahidol University, Bangkok, Thailand; 4 Division of Gastroenterology and Hepatology, Department of Medicine, University of Alabama at Birmingham, Birmingham, AL

**Keywords:** GI bleed, small intestine, Turner syndrome, female

## Abstract

Small intestinal venous abnormalities are an underrecognized condition as an etiology of overt gastrointestinal (GI) bleeding in patients with Turner syndrome. Evidence-based therapeutic options for these lesions are lacking in the published literature. A 47-year-old woman with Turner syndrome with a 30-year course of recurrent GI bleeding was found to harbor diffuse small intestinal venous ectasias through endoscopic imaging. Achievement of a 21-month clinical remission (elimination of hospitalizations for overt GI bleeding and normalization of hemoglobin concentration) was reached after initiation of tranexamic acid titrated to 2,600 mg daily.

## INTRODUCTION

Turner syndrome (TS) is a sex chromosomal disorder with an estimated prevalence of 1 in 2,000 women. Partial or complete reduction in the X chromosome has long been described to be causal of multiple health issues including but not limited to webbed neck, short stature, skeletal abnormalities, congenital heart defects, endocrinopathies, and autoimmune disorders. A population-based study demonstrated a 13-fold increased risk of liver disease and 3-fold increased risk of gastrointestinal (GI) hemorrhage in TS.^1^ There seems to be an increased risk of inflammatory bowel disease and a distinct predisposition to GI bleeding from GI angioectasias and venous anomalies throughout the GI tract. This GI bleeding predisposition in TS has been underrecognized in the medical literature because there was a total of 41 cases reported in 2018.^2^ Therapy for recurrent GI bleeding in TS may be endoscopic, angioembolic, or surgical when it can be localized but presents a conundrum when diffuse in character. We report a case of occult and overt melenic GI bleeding over several decades from diffuse small intestinal venous anomalies in a patient with TS who demonstrated a dramatic clinical response to tranexamic acid (TXA).

## CASE REPORT

**Figure 1.**
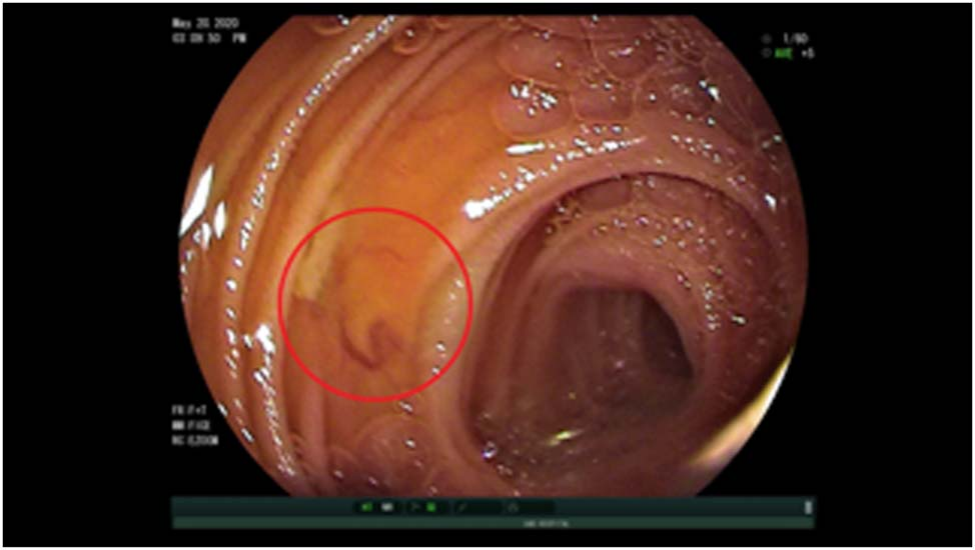
Gastrointestinal venous ectasias demonstrated by capsule endoscopy in 2011 (red circle).

A 47-year-old woman with TS, a history of partial anomalous pulmonary venous return and bicuspid aortic valve, presented with recurrent melenic stool and iron deficiency anemia since 15 years of age. There was a long smoking history but no history of aspirin or nonsteroidal anti-inflammatory use. Multiple endoscopic and radiologic evaluations elsewhere had been nondiagnostic. On our initial evaluation, esophagogastroduodenoscopy, colonoscopy, computed tomography enterography, computed tomography angiography, and Meckel scan were nondiagnostic. Initial video capsule endoscopy (VCE) in 2010 was negative, but subsequent overt bleeding event led to VCE in 2011 with findings of multiple dilated venous anomalies most notably in the ileum (Figure 1). A trial of somatostatin analog therapy monthly was initiated, but the patient was subsequently lost to follow-up.

**Figure 2.**
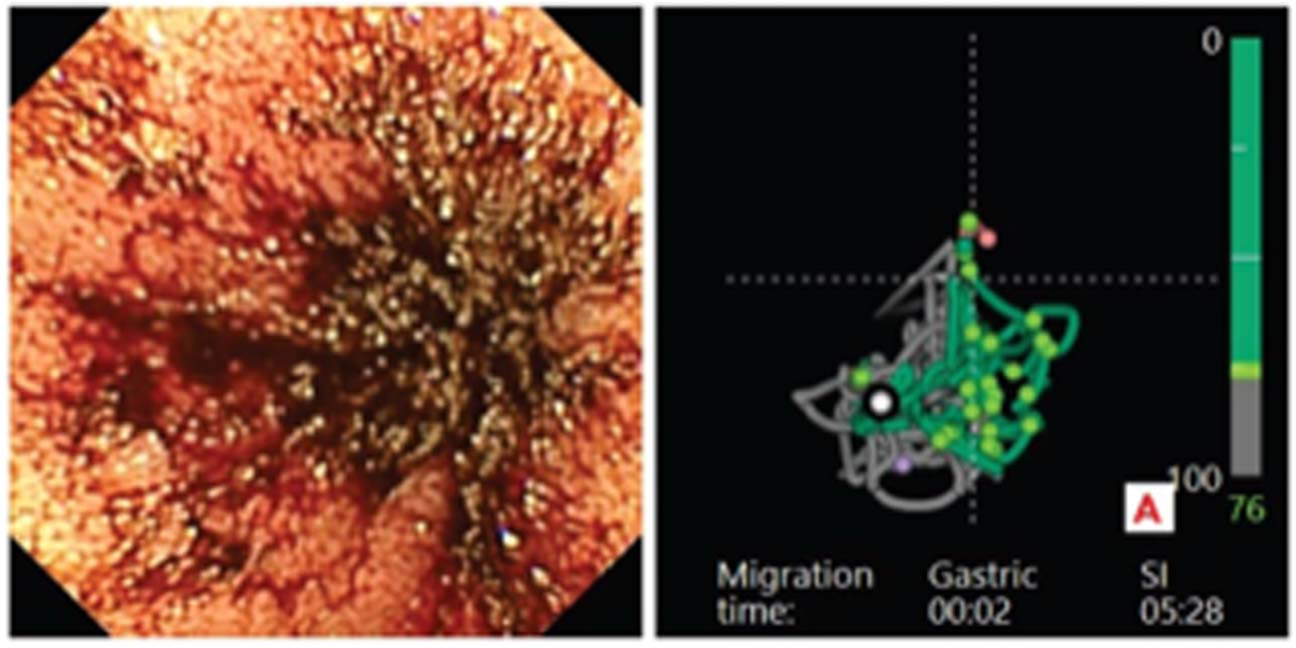
Active bleeding in the mid-small bowel demonstrated by capsule endoscopy at the 04:12:15 mark time in 2020.

She re-established care with us in April 2017 when she presented with a 3-day history of dark stool and a hemoglobin of 6.7 g/dL. She reported multiple hospitalizations requiring transfusions, parenteral iron, and nondiagnostic endoscopic procedures at other institutions from 2011 to 2017. Recurrent melenic stool and/or severe symptomatic anemia led to 12 hospital admissions and 18 units of packed red blood cell transfusions between 2017 and 2019. Balloon-assisted deep enteroscopy was nondiagnostic from the anterograde approach, but VCE in April 2020 when presenting with hemoglobin 3.5 g/dL showed active bleeding in the mid-small bowel (Figure 2). Repeat balloon-assisted deep enteroscopy revealed many nonbleeding dilated jejunal and ileal veins felt to be too numerous for endoscopic therapy.

**Figure 3.**
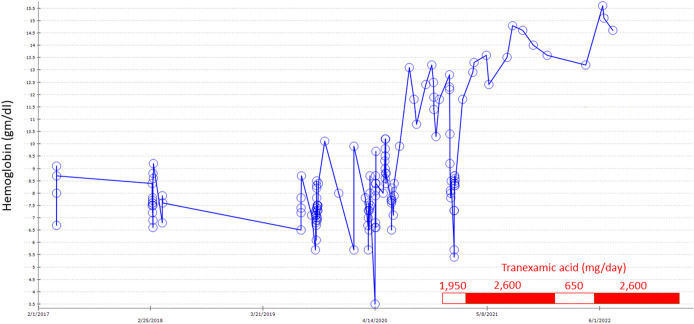
Graph illustrating the hemoglobin level from 2017 to 2022 with illustration of tranexamic acid initiation and dosing.

Previous failure of somatostatin analog therapy and long-standing smoking history precluding safe estrogen/progesterone therapy led to the initiation of TXA 650 mg orally TID in December 2020. She was readmitted with melenic bleeding 24 days later with nadir hemoglobin of 8.5 g/dL. TXA was held throughout that 9-day hospitalization but restarted at discharge. She presented again in January 2021 with melena and hemoglobin of 5.7 g/dL and required further packed red blood cell transfusion. TXA dose was increased to 2,600 mg/d TID after that admission. Subsequently, she has not required hospital admission for GI bleeding or anemia over the ensuing 21 months, and hemoglobin has shown a sustained normalization from the previous low baseline (Figure 3). TXA dose was uneventfully temporarily reduced to 650 mg daily in early 2022 for a series of oropharyngeal surgeries.

## DISCUSSION

TS is a well-known chromosomal disorder first recognized by an Oklahoma physician Dr. Henri Turner in 1938. Multiple phenotypes were reported to be associated with the “congenital ovarian dysgenesis” syndrome, and GI arteriovenous anomalies were estimated to be found in 7% in the early literature.^3^ The true prevalence of GI anomalous vascular malformations remains uncertain because karyotyping to confirm the diagnosis has not been uniformly performed. The uncertainty of the true prevalence of luminal vascular anomalies in TS and their predilection for the small intestine suggests that clinical gastroenterologists consider VCE when bidirectional endoscopy is nondiagnostic in the setting of occult or overt GI bleeding in patients with TS and that repeat VCE may sometimes be required for accurate diagnosis.

Therapeutic options for TS patients with symptomatic GI vascular anomalies are not well-studied and mirror the therapeutic approach for angioectasia therapy in patients without TS. Endoscopic, angioembolic, or surgical therapy for localized lesions is straightforward. Medical therapy is often required for diffuse vascular lesions and may include somatostatin analogs, estrogen/progesterone therapy, thalidomide, or bevacizumab.^4^ The unusual venous anomalies in patients with TS may suggest a different kind of endothelial defect than that found in the more widespread problem of angiodysplasia which have an arteriolar component.^5^ It is unclear if this may translate to differential therapeutic responses.

TXA is a synthetic derivative of the amino acid lysine that exerts an antifibrinolytic effect through a reversible blockade of lysine binding sites on plasminogen molecules. It has a favorable side effect profile and has been used to control postoperative bleeding, epistaxis, menorrhagia, and intracranial hemorrhage. Whether the therapeutic benefit in our case is due to fibrinolysis inhibition, venous epithelial stabilization, or another mechanism is unclear. A recent meta-analysis in emergent GI bleeding suggested benefit with the use of TXA regarding mortality, urgent endoscopic intervention, and ongoing bleeding.^6^ The optimal dose of TXA therapy in GI bleeding remains inconclusive. A high-dose approach of 3.9 g daily was effective in a case of refractory GI bleeding (from jejunal and colonic angioectasias) in a patient who had failed endoscopic and somatostatin analog therapy.^7^ In our patient, low-dose TXA seemed ineffective initially, but higher dose therapy led to an ongoing 21-month cessation of overt melenic GI bleeding, hospitalizations, and normalization of previously significant iron deficiency anemia suggesting a possible dose-dependent effect. This is the first report in the literature of effective, long-term TXA therapy for small intestinal venous anomaly bleeding in TS. The ease of administration makes it an attractive option in this setting. Further clinical trials and case series would seem warranted to fully establish the efficacy and optimize the dosing of this therapeutic option.

## DISCLOSURES

Author contributions: All authors granted the approval of the manuscript to be published. F. Weber acquired the data and is the article guarantor. P. Lertwilaiwittaya drafted the initial manuscript. All authors performed critical review and edited the manuscript.

Financial disclosure: None to report.

Informed consent was obtained for this case report.
